# Tuning the Size of Thermoresponsive Poly(*N*-Isopropyl Acrylamide) Grafted Silica Microgels

**DOI:** 10.3390/gels3030034

**Published:** 2017-09-17

**Authors:** Nils Nun, Stephan Hinrichs, Martin A. Schroer, Dina Sheyfer, Gerhard Grübel, Birgit Fischer

**Affiliations:** 1 Institute of Physical Chemistry, University of Hamburg, 20146 Hamburg, Germany; nils.nun@studium.uni-hamburg.de (N.N.); Stephan.Hinrichs@chemie.uni-hamburg.de (S.H.); 2 Deutsches Elektronen-Synchrotron DESY, Notkestr. 85, 22607 Hamburg, Germany; mschroer@embl-hamburg.de (M.A.S.); dina.sheyfer@desy.de (D.S.); gerhard.gruebel@desy.de (G.G.); 3 The Hamburg Centre for Ultrafast Imaging (CUI), Luruper Chaussee 149, 22761 Hamburg, Germany

**Keywords:** hydrogel, pNipam, core-shell particle, lower critical solution temperature (LCST), thermoresponsive

## Abstract

Core-shell microgels were synthesized via a free radical emulsion polymerization of thermoresponsive poly-(*N*-isopropyl acrylamide), pNipam, on the surface of silica nanoparticles. Pure pNipam microgels have a lower critical solution temperature (LCST) of about 32 °C. The LCST varies slightly with the crosslinker density used to stabilize the gel network. Including a silica core enhances the mechanical robustness. Here we show that by varying the concentration gradient of the crosslinker, the thermoresponsive behaviour of the core-shell microgels can be tuned. Three different temperature scenarios have been detected. First, the usual behaviour with a decrease in microgel size with increasing temperature exhibiting an LCST; second, an increase in microgel size with increasing temperature that resembles an upper critical solution temperature (UCST), and; third, a decrease with a subsequent increase of size reminiscent of the presence of both an LCST, and a UCST. However, since the chemical structure has not been changed, the LCST should only change slightly. Therefore we demonstrate how to tune the particle size independently of the LCST.

## 1. Introduction

There is a large number of hydrogels that responds to external stimuli like temperature and pH [1,2,3]. These so called stimuli-responsive hydrogels offer a broad range of applications in the biomedical (e.g., controlled drug delivery systems [4]) as well as technical fields (e.g., in catalysis [2,5]). One of the most investigated stimuli-responsive hydrogels is poly(*N*-isopropyl acrylamide) (pNipam) due to its lower critical solution temperature (LCST) in aqueous solution of about 32 °C [2,6]. Being close to human body temperature, it is an ideal candidate for biomedical applications [7]. Above the LCST pNipam expels water and undergoes a coil-to-globule transition. This is similar to the cold denaturation of proteins [8], and can therefore be used as a model system for proteins to study the influence of osmolytes [9,10]. An upper critical solution temperature (UCST) in aqueous solution is less common for polymers. With a UCST polymers are solubilized above the UCST in aqueous solution [11]. The change of the solution behaviour depends on the hydrophobic nature of the solvent. pNipam exhibits a UCST behaviour using a solvent mixture like water/ethanol or water/dimethyl sulfoxide with a low water content [12,13].

One synthesis method for the preparation of pNipam is the free radical emulsion polymerization [6]. Sodium dodecyl sulphate (SDS) can be used as surfactant and methylene-*bis*-acrylamide (BIS) as crosslinker connecting two pNipam chains. By switching the temperature the pNipam chains elongate below the LCST and make a coil-to-globule transition above the LCST. Without the crosslinker, the pNipam chains randomly form globular structures above the LCST to minimize their contact with water. In presence of the crosslinker the coil-to-globule-transition of the pNipam chains gets reversibly. The addition of crosslinker increases the LCST slightly [14]. The crosslinker itself has a higher reaction rate than *N*-isopropyl acrylamide (Nipam) [5], therefore most of the prepared microgels have a heterogeneous distribution of the crosslinker within the pNipam network decreasing from the inside to the outside of the microgel [5,15,16,17]. This distribution or internal structure has a high impact on the swelling behaviour of the microgel [18], that is, the degree of swelling of a microgel with homogeneously distributed crosslinker is higher than the degree of swelling of a microgel with heterogeneously distributed crosslinker [18]. 

A disadvantage of pNipam microgels is its relatively high softness in the swollen state which limits the mechanical stability. Interpenetrating networks of different polymers or incorporated nanoparticles have been created to make pNipam hydrogels more interesting in terms of mechanical investigations [1]. In particular, silica nanoparticles are of special interest as these can be easily and highly reproducibly synthesized. In addition, the synthesis can be directly used for nanoparticles covered with a silica shell, which is an frequently used method [19]. By incorporating nanoparticles inside the silica core, multi-responsive hydrogels can be prepared with additional optical or magnetic properties [20,21,22,23,24,25].

Several methods exist to synthesize core-shell particles containing a silica core and a pNipam shell [26,27]. Here, we report on a synthesis route for pNipam core-shell microgel by controlling the growth process of the pNipam shell together with the crosslinker BIS on the silica surface. We show that depending on the growth process we can alter the thermo-responsive properties of the pNipam shell. The pNipam microgel seems to behave like it has either an LCST, a UCST, or even both. However, since the chemical structure has not been changed compared to the literature [5,28], pNipam should still have an LCST. This means by altering the synthesis route slightly we control the internal structure of the pNipam shell, and with this we can tune the hydrodynamic size independently from the coil-to-globule transition of pNipam.

## 2. Results and Discussion

To demonstrate the three different scenarios of thermal responsivity described above we will show one example for each case. Each system consists of a silica core and a pNipam shell. Due to the difference in the synthesis procedures the distribution of the crosslinker densities differs in each system. For each synthesis a new core was synthesized. First we discuss the silica core for each system. Then we compare the different microgel systems. 

### 2.1. Characterization of the Silica Core

**Figure 1:**
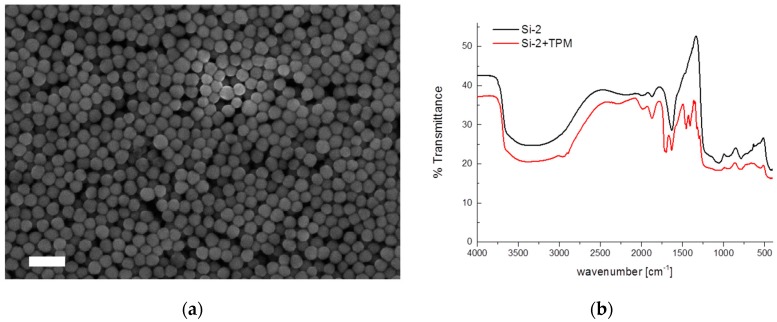
(**a**) Scanning electron microscopy (SEM) image of silica particles (Si-2). The scale bar is 200 nm; (**b**) Fourier transformed infrared spectroscopy (FTIR) spectra of Si-2 before and after (Si-2+TPM) surface modification with TPM.

For each microgel a silica core has been synthesized via the Stöber process. The Stöber process results in spherical particles [29]. The hydrodynamic sizes of the silica particles were measured by dynamic light scattering. For this, a dilute sample of each silica core was measured at 25 °C. The samples Si-1–Si-3 have a radius of 67 nm, 31 nm, and 36 nm, respectively. Exemplarily for sample Si-2, the scanning electron microscopy (SEM) image is shown (Figure 1a).

To link the pNipam onto the particle surface, the silica surface was modified with 3-(trimethoxysilyl)-propyl-methacrylate (TPM). The addition of TPM does not affect the size of the particles much. However, it does change the surface properties and make them more hydrophobic. After the successful addition of TPM the particles are no longer dispersible in water, but they can be well dispersed with ethanol. In addition it allows for chemical coupling between the silica surface and the pNipam chains [26]. Thermogravimetric analysis shows (Figure S1 and Table S1), that a small organic content is present in the sample compared to pure silica particles. Additionally, Fourier transformed infrared spectroscopy (FTIR) confirms the presence of TPM bound to the silica surface (Figure 1b). From the silica a broad and almost featureless band is visible between 2500 and 3800 cm^−1^, corresponding to the stretching vibrations of the surface silanols Si-OH perturbed by hydrogen, bonding either intramolecularly or with adsorbed water [30]. For the TPM-modified silica particles, additionally two small bands are visible between 2800 and 3000 cm^−1^ from the stretching vibration of C-H bonds. The bands at 1710, 1450 and 1410 cm^−1^ correspond to the stretching vibration of C-O, methylene C-H and vinyl C-H bending vibration of TPM [31]. 

### 2.2. Temperature Behaviour of the Hybrid Hydrogel

#### 2.2.1. Sample SiPN-1

**Figure 2:**
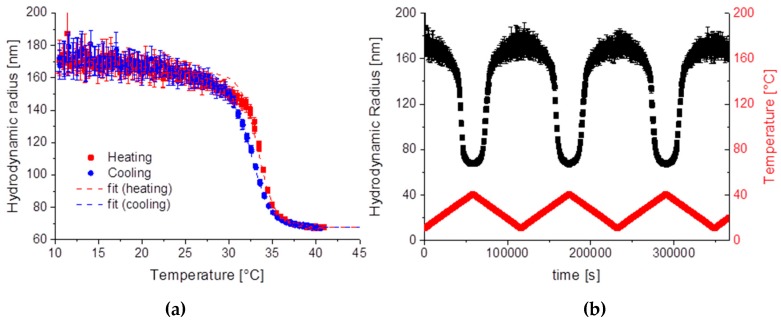
(**a**) Hydrodynamic radius of SiPN-1 for a heating (black cubes) and cooling cycle (red dots) with an empirical fitting function Equation (1); (**b**) Hydrodynamic radius for several heating and cooling cycles for SiPN-1. In red the temperature gradient is shown.

For all three samples the pNipam shell was made out of Nipam/BIS with about 3–4 mol % BIS, for SiPN-1 about 3.2 mol % was used. In case of SiPN-1 both monomers are added at the beginning of the polymerization process. Pure pNipam has an LCST of about 32 °C. By using a copolymerization with BIS the LCST can be slightly shifted to higher temperatures. The SiPN-1 particles collapse above a temperature of about 33.2 °C during heating and swell below 32.4 °C during cooling (Figure 2a). The LCST T* is determined by fitting the data with a Boltzmann function [32]:

*R*_min_ is the minimum hydrodynamic radius and was about 68 nm for heating and cooling, which is close to the diameter of the silica core, which is about 67 nm (Si-1). The LCST T* is at about 33.4 ± 0.1 °C (32.3 ± 0.1 °C) for heating (cooling). The swelling and deswelling behaviour are reversible as is found by cycling the sample (Figure 2b).

(1) $$R_{\mathrm{hyd}}=\frac{R_{\min}-R_{\max}}{1+\exp\left(-\frac{T-T^{*}}{dT}\right)}$$

**Figure 3:**
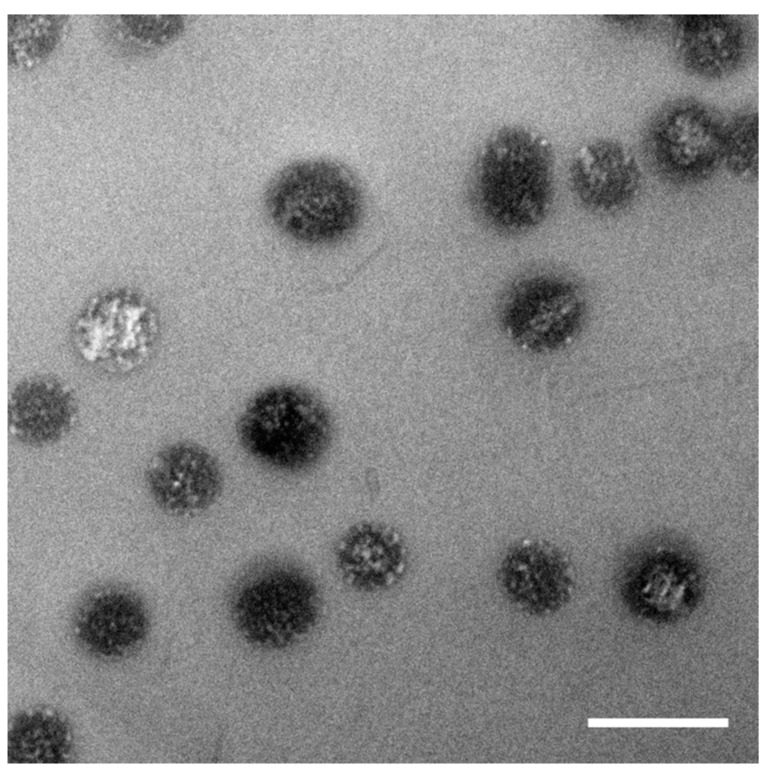
TEmicrograph of SiPN-1. Scale bar is 200 nm. Each particle has a darker core and a rough surface resulting from the dried microgel shell.

In all dynamic light scattering data only a single decay has been observed, this is a clear indication of just one particle species. For the Transmission electron microscopy (TEM) measurements, the particles have been dried on a carbonated copper grid. By this preparation method for TEM the particles resemble the state above the LCST. In this phase the particles are collapsed and all water is squeezed out of the microgel particles. A darker core—which results from the silica core—and a bright corona—which corresponds to a thin rough surface—are visible in TEM (Figure 3). No pure pNipam particles are visible in the TEmicrograph. 

#### 2.2.2. Sample SiPN-2

While for SiPN-1 the crosslinker and Nipam were added simultaneously, for SiPN-2 the whole amount of crosslinker BIS was added at the beginning of the encapsulation, followed by Nipam. The molar ratio of the crosslinker was about 4 mol %, a little higher amount than for SiPN-1. The pNipam chains at the surface of the silica particles are more densely crosslinked than at the periphery. Compared to the reaction of SiPN-1, the initiator concentration was reduced as well as the redoxsystem concentration. The SDS concentration as well as the initiator concentration was the same like for SiPn-1.

**Figure 4:**
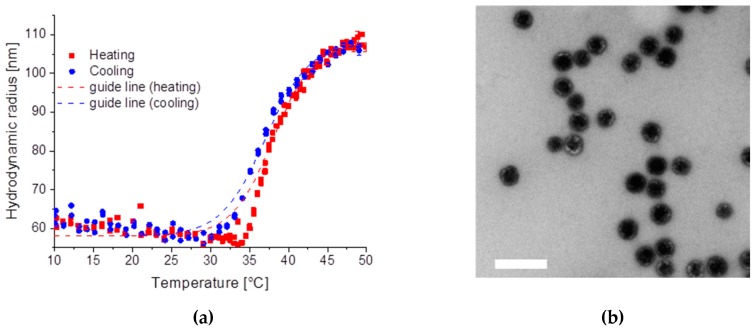
(**a**) Hydrodynamic radius of SiPN-2 as a function of temperature for heating and cooling; (**b**) TEmicrograph of SiPN-2. Scale bar is 200 nm.

Like for SiPN-1 neither pure pNipam particles are visible in TEM nor a second decay could be observed by dynamic light scattering. The hydrodynamic radius of the microgel particles increases from 58 nm to about 108 nm by increasing the temperature (Figure 4a). The guide line for cooling and heating was done by Equation (1). Here the core size of the silica particles (Si-2) is 31 nm, which results in a pNipam shell of at least 30 nm. The transition temperature was about 38.0 °C for heating and 37.0 °C for cooling. The TEmicrograph shows a similar picture like for SiPN-1, a darker image for the silica core and a brighter surface corona in the collapsed state (Figure 4b).

#### 2.2.3. Sample SiPN-3

For the third sample the monomers Nipam and BIS were grown in three steps, which results in three shells with a heterogeneous distribution relatively to sample SiPN-1; therefore, the density of the crosslinker always decreases from the inside to the outside within a shell. The molar ratio of the crosslinker was ~3.2% for the first two shells, similar to SiPN-1 and 3.7% for the third shell, which was similar to SiPN-2. For the synthesis the same concentration of SDS, Na_2_SO_3_, K_2_S_2_O_8_ and (NH_4_)_2_Fe(SO_4_)_2_ as for SiPN-1 were used. The reaction temperature was lowered to 50 °C, which slows down the reaction compared to 60 °C.

**Figure 5:**
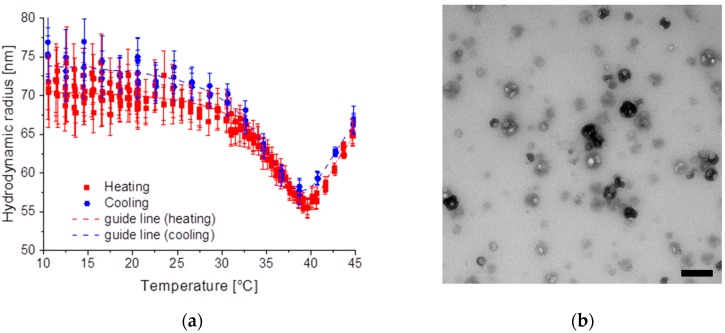
(**a**) Hydrodynamic radius of SiPN-3 as a function of temperature for heating and cooling; (**b**) TEmicrograph of SiPN-3. Scale bar is 200 nm.

Measuring the temperature-dependent hydrodynamic radius of the microgel particles shows a scenario such as for the presence of both an LCST as well as a UCST at higher temperatures as the hydrodynamic radius exhibits a minimum size (Figure 5a). Additionally, here only a single decay has been observed by dynamic light scattering, which indicates only one particle size. The minimal hydrodynamic radius is about 56 nm for heating at 39.6 °C and, for cooling, 58 nm at 38.6 °C. The silica core (Si-3) has a diameter of about 36 nm which results in a pNipam shell size of at least 20 nm. In the TEM images darker spheres show the electron denser silica particles which have some brighter spheres on their surface, which result from the collapsed pNipam chains (Figure 5b). 

However, it is also visible that due to the drying process agglomerates are formed which have not been observed in the dynamic light scattering experiments, and which are very sensitive to large particles.

### 2.3. Discussion

Below, the LCST the pNipam hydrogel swells with water and the pNipam chains tend to elongate. At temperatures above the LCST pNipam gets more hydrophobic and expels water. Due to the surface charge resulting from the initiator, the pNipam is still dispersible in water and does not precipitate. This is the usual behaviour of pNipam microgel dispersions. Sample SiPN-1 shows this typical behaviour of pNipam microgels (Figure 2a). At low temperatures the hydrodynamic radius is larger than at high temperatures because the particle is swollen with water. Above the LCST the particle radius shrinks due to the squeezing out the water. This temperature behaviour is repeatable over several cycles (Figure 2b). TEM shows a thin pNipam skin of less than 5 nm covering the silica particles (Figure 3). The microgel particles in the collapsed state have a similar hydrodynamic radius as the pure silica particles in accordance with this observation by TEM.

Since Si-1 has twice the radius of Si-2, a smaller amount of Nipam was used for the synthesis to reach a similar shell thickness. In addition, a lower amount of initiator was used. This leads, on the one hand, to lower surface charges and, on the other hand, to fewer but longer pNipam chains. Thus, SiPN-2 shows a completely different temperature scenario. By increasing the temperature the hydrodynamic radius increases, resembling the presence of a UCST, although pNipam still becomes more hydrophobic, which is visible on the changing turbidity. At high temperature the sample SiPN-2, as well as SiPN-3, turns turbid as a result of the change in the refractive index of the microgel after squeezing out the water. Here, the minimum radius of the microgel particles reaches about 60 nm and is larger than the one of the pure silica particle with a hydrodynamic radius of about 31 nm (Si-2). In contrast, in TEM the sample does not look different to SiPN-1, only the pNipam shell seemed to be thicker (~30 nm). 

SiPN-3 shows a scenario like for the presence of both an LCST as well as a UCST. A possible explanation is visible in the TEmicrograph (Figure 5b). Here, instead of a rough surface, microgel balls are visible. Since the hydrophobicity of the pNipam chains increases at high temperature a spherical form is advantageous because of its minimized surface area and with it the contact to water. Several studies of pNipam grafted silica particles or gold nanoparticles demonstrate that pNipam does not change its chemical behaviour due to grafting and still has an LCST around 32 °C [22,27,28,33,34,35,36,37]. Variation of the crosslinker density only has an effect on the swelling degree and the LCST temperature [14], however, not on the solvation behaviour. So far only through the copolymerization of Nipam with a small amount of methacrylic acid and nitrocatechol monomers in phosphate-buffer in saline medium was UCST behaviour found [38]. In addition, by adding salts of the Hofmeister series [39] and by using mixtures out of water and co-non-solvents like ethanol, the thermo-responsive behaviour changes [12,13,40,41]. 

All SiPN samples studied here were dialysed against water, so no salt or any other solvents were present during the measurement. Thus, the observed response is not due to any additional solute. The organic content lies for all three systems at about 70–80 wt % (see TGA measurements Figure S1 and Table S1). Therefore, we suggest that the difference in the temperature behaviour occurs due to the internal structure of the microgel. 

**Figure 6:**
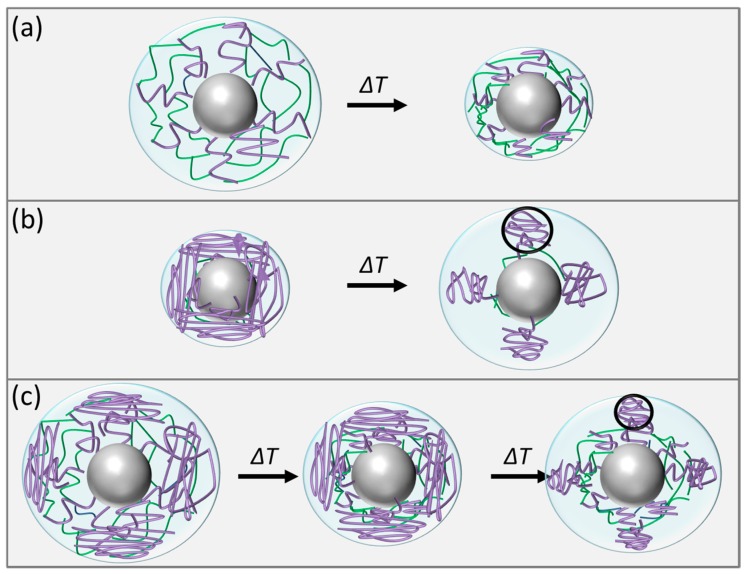
Different scenarios for the core-shell particles during the coil-to-globule transition. The silica core is shown as a grey sphere. The poly-(*N*-isopropyl acrylamide) (pNipam) chains are represented in purple and the crosslinking chains between two pNipam chains are visualized in green. The light blue circles indicate the hydrodynamic volume of the particles. Scenario (**a**): Below the lower critical solution temperature (LCST): The microgel particle is swollen with water and the pNipam chains are elongated. The pNipam chains are crosslinked from the inner to the outer shell. Above the LCST the pNipam shell collapses. Due to the internal crosslinking the pNipam shell homogeneously shrinks and the pNipam shell gets thinner due to the expulsion of water. Scenario (**b**): Below the LCST the pNipam chains are elongated and linked to the silica surface. Only near the surface of the silica particles are the pNipam chains crosslinked. Therefore, above the LCST the pNipam chains collapse into small spheres onto the surface of the silica particles. These small spheres we call microgel balls—we highlighted one with a black circle. Scenario (**c**): Below the LCST the microgel is swollen with water and the pNipam chains are elongated. By increasing the temperature the microgel structure expels water and shrinks. Due to external crosslinking the shell gets thinner like in scenario (**a**). However, since long not-crosslinked pNipam chains are also presented, these pNipam chains show up as microgel balls on the surface.

To understand why the samples SiPN-2 and SiPN-3 show a behaviour which can be compared to a UCST behaviour, we tried to sketch the different scenarios in Figure 6. The pNipam chains of sample SiPN-1 are well crosslinked (Figure 6a). Due to this internal crosslinking the pNipam shell homogeneously shrinks and the pNipam shell gets thinner due to the expulsion of water and the hydrodynamic radius decreasing at higher temperature. The pNipam hydrogel in SiPN-2 is only densely crosslinked near the surface of the silica particles (Figure 6b). Due to the addition of BIS only at the beginning of the synthesis, the crosslinker concentration decreases, which means that single pNipam chains grow from a thin crosslinked pNipam shell on the silica surface. This makes it possible that the pNipam chains stay at the silica particle. The lower amount of the initiator used for SiPN-2 also favours the growth of long chains. If the pNipam chains collapse, these chains form a globular structure on the surface of the silica particles (Figure 6b). Since spheres have the smallest surface area they form microgel balls (highlighted by a black circle in Figure 6b) onto the surface to minimize the contact with water. This leads to an increase of the hydrodynamic radius in total. It might also explain the thicker pNipam shell of SiPN-2 at high temperatures compared to SiPN-1, because here, instead of a thin homogeneous pNipam shell, several thick microgel balls are present. However, since the microgel balls seem to cover the complete surface these are not clearly visible in TEM (Figure 4b).

For the last sample the pNipam network is not as crosslinked as in the first sample SiPN-1, but also not as loose as for sample SiPN-2, where the pNipam chains are only crosslinked at the silica surface. Here it seems both scenarios play a role (Figure 6c). 

In this case first the crosslinked pNipam chains collapse and expel water, the size of the shell decreases. However, since not all chains are crosslinked, the pNipam chains build microgel balls linked to the silica surface. This is clearly visible in the TEmicrogaph (Figure 5b). 

**Figure 7:**
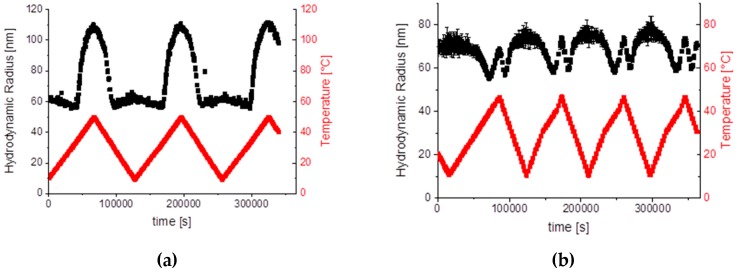
Hydrodynamic radius for several heating and cooling cycles for sample SiPN-2 (**a**) and SiPN-3c (**b**).

The temperature behaviours for SiPN-2 and SiPN-3 are repeatable; the hydrodynamic radii were measured for both samples for several heating and cooling cycles (Figure 7).

## 3. Conclusions

Three different pNipam grafted silica microgels have been prepared. In general the synthesis can be divided into three steps [26]. First the silica core is synthesized by a modified Stöber process; second, the surface was modified by TPM; third, silica cores were grafted with pNipam.

By changing the way of adding the crosslinker the temperature-responsive behaviour can be tuned. Three different temperature scenarios have been detected. The first scenario has the typical behaviour of pNipam with an LCST temperature of about 33.4 ± 0.1 °C (32.3 ± 0.1 °C) for heating (cooling). The second resembles a USCT-like behaviour. 

In the last scenario both LCST and UCST-like behaviour seem to be combined. However, the chemical composition has not been changed compared to other studies [22,27,33,34,35,36,37], therefore pNipam should still have an LCST temperature. However, the swelling and collapsing has the opposite effect on the hydrodynamic radius due to the formation of pNipam microballs on the surface of the silica particles. 

The incorporating of a silica core helps to improve the mechanical properties of pNipam. Growing pNipam onto a silica surface makes it possible to tune the hydrodynamic particle radius differently. This enables us to tune the particle size independently of the swelling and deswelling behaviour.

## 4. Materials and Methods

### 4.1. Materials

Tetraethylorthosilicate (TEOS) for synthesis (≥99%), potassium peroxodisulphate (K_2_S_2_O_8_) for analysis (≥99%), sodium sulphite (Na_2_SO_3_) for analysis (≥97%), and *N*,*N*′-methylenebisacrylamide (BIS) for synthesis (≥98%) were purchased from Merck (Darmstadt, Germany).

3-(trimethoxysilyl)-propyl-methacrylate (TPM) (≥98%) was purchased from Aldrich (Munic, Germany).

Ethanol (99.5%, dried over molecular sieves) and *N*-isopropyl acrylamide (Nipam) (stabilized, 99%) were purchased from ACROS Organics (Nidderau, Germany). 

Ammonium hydroxide (NH_3_; 25%) for analysis was purchased from VWR Chemicals (Darmstadt, Germany).

Sodium dodecyl sulphate (SDS) for biochemistry (≥99%) was purchased from Carl Roth GmbH (Karlsruhe, Germany).

Ammonium iron (II) sulphate hexahydrate ((NH_4_)_2_Fe(SO_4_)_2_) American Chemical Society (ACS) reagent (99%) and technical ethanol (92.6–93.8%) were purchased from Sigma Aldrich (Munic, Germany). The technical ethanol was distilled twice prior to use. The other chemicals were used as obtained. 

### 4.2. Synthesis

The synthesis method of silica particles grafted with pNipam can be divided into three steps. First, a silica core is prepared, and then a methacrylate group is grafted onto the surface of the silica particles, which finally could be used as a starting point for growing of the pNipam/BIS chains. The different synthesis steps are given in detail below.

#### 4.2.1. TPM-Grafted Silica Particles

The synthesis of silica particles is derived from the Stöber-process [29]. Ethanol was mixed with ammonium hydroxide (A, Table 1) and stirred for a few minutes. TEOS was added, followed by substantial stirring. After another 12 h of stirring additional ammonium hydroxide (B, Table 1) was added together with TPM similar to the synthesis reported by Karg et al. [26]. Afterwards, the ethanol/ammonium hydroxide was removed until only a few millilitres of ethanol were left. The sample was kept dispersed with ethanol and dialyzed against ethanol to remove the residual TPM. The specific amounts are displayed in Table 1.

#### 4.2.2. pNipam-Grafted Silica Microgels

The pNipam shell was synthesized similar to Karg et al. [26]. In a typical synthesis SDS and Na_2_SO_3_ were dissolved in deionized water in a three neck round flask. TPM-grafted silica particles were added and have been stirred for one hour under nitrogen atmosphere. Potassium peroxodisulphate was added as initiator and one crystal of ammonium iron (II)-sulphate, which acts together with Na_2_SO_3_ as a redox-system to decrease the reaction temperature. 

To change the internal structure of the microgel shell the method to add Nipam and BIS was varied, that is, the order of the monomers and the addition rate as well as the reaction temperature.

The microgel particles were directly dialyzed against deionized water for a week to remove the excess of SDS. Then, the particle dispersions were concentrated with a rotary evaporator under reduced pressure until only a few millilitres were left. The samples were always kept in water. For purification the concentrated dispersions were dialyzed again against water for several days and filtered afterwards.

To ensure that pNipam/BIS grow onto the surface of the silica particles diluted dispersions, especially low-monomer concentrations are used. In this case the probability for the monomers to find each other to build additional pNipam particles is reduced. This effect can be further reduced by pumping the monomer to the dispersions. In case of unwanted side products, pure pNipam particles are visible in TEM and can be observed as a second correlation peak by dynamic light scattering (DLS). 

##### SiPN-1

Si-1 was used as the silica core. The sample was diluted with water to 1.5 L. Afterwards, the addition of Nipam and BIS was done in two steps. First 44.19 mmol Nipam and 1.43 mmol BIS (3.2 mol %) were added. After one hour of stirring another 44.19 mmol Nipam and 1.43 mmol BIS (3.2 mol %) were added. The reaction has been left stirring for six hours at 60 °C. The used amounts of the chemicals are 0.87 mmol/L SDS, 2.20 mmol/L Na_2_SO_3_, 1 mmol/L K_2_S_2_O_8_, and 1 crystal (NH_4_)_2_Fe(SO_4_)_2_.

##### SiPN-2

Si-2 was used as the silica core and diluted to a volume of about 1.5 L with water. The addition of 20 mMol Nipam and 0.80 mmol BIS (4 mol %) was done by pumping with a pumping speed of 1 mL/min. For this purpose, BIS was dissolved in 10 mL ethanol and Nipam was dissolved in 40 mL ethanol. After 5 min of pumping the monomers into the solution, the influx was stopped for 15 min, and afterwards this process was repeated until all monomer was inserted. At the beginning only BIS was added. After 40 min the Nipam was pumped to the reaction. 

The specific amounts of each chemical are 0.88 mmol/L SDS, 1 mmol/L Na_2_SO_3_, 0.25 mmol/L K_2_S_2_O_8_ and 1 crystal (NH_4_)_2_Fe(SO_4_)_2_. The reaction has been stirred at 60 °C for about 15 h. 

##### SiPN-3

Si-3 was used as the silica core. The addition of Nipam and BIS was done in three steps; the specific amounts of each chemical are displayed in Table 2. Each time the synthesis procedure was repeated and the mixture has been stirred at 50 °C for about 6 h. During the reaction the SDS concentration was kept constant at 0.87 mMol/L, the Na_2_SO_3_ at 2.2 mmol/L and the K_2_S_2_O_8_ at 1 mmol/L.

### 4.3. Characterization Methods

Dynamic light scattering (DLS) measurements were conducted on an ALV/CSG-3 Compact Goniometer-System using an ALV/LSE-5003 Multiple Tau Digital Correlator working with pseudo cross-correlation and the ALV Digital Correlator Software 3.0. The measuring angle was set to 90° for all measurements. As a light source, an Nd:YAG laser emitting at 532 nm was used. A toluene bath which was used for matching of the index of refraction was tempered by a Julabo F25 thermostat working with a mixture of water and ethylene glycol and delivering a temperature accuracy of 0.01 °C. For the measurements diluted samples were used and the temperature was varied between 10 and 45 °C (50 °C for SiPN-2). Around the LCST transition the temperature was increased in 0.5° steps above 40 °C and below 30 °C in 1 °C steps. Each temperature was measured at least three times. After each temperature was reached, the sample has been given 3 min time to equilibrate. The acquisition time was 120 s for sample SiPN-1, 60 s for SiPN-2 and SiPN-3 per temperature step. During the cooling cycle less temperature steps were done. Prior to the measurement all samples were diluted to have a volume fraction of about 0.1%. Below the LCST the samples were clear dispersions. Above the LCST the dispersions were a slightly turbid.

Transmission electron microscopy (TEM) was performed on a Philips CM-300 microscope operating at 300 kV and on a FEI Tecnai 12 transmission electron microscope operating at 120 kV. TEM samples were prepared by dropping diluted solutions transferred in ethanol onto a 400-mesh carbon coated copper grid with the excessive solvent being immediately evaporated.

Scanning electron microscopy (SEM) was obtained on a high resolution SEM (Gemini Leo 1550) at an acceleration voltage of 5 keV. SEM samples were prepared by dropping diluted solutions on a silicon wafer. The excessive solvent was evaporated afterwards. 

Thermogravimetric analysis (TGA) was conducted on a Netzsch TGA 209 F1 Iris. The experiments were run with a heating rate of 10 K/min in a range of 25–500 °C and conducted in an inert nitrogen atmosphere. Therefore, about 6–8 mg of the specimen was weighed into an 85 μL aluminium oxide crucible (purchased from Netzsch, Selb, Germany). The data processing was performed by Netzsch Proteus Software afterwards (4.7.0, Selb, Germany).

Fourier Transform infrared spectra were performed on a VERTEX-70/FT-IR spectrometer (Bruker Optics, Ettlingen, Germany). Therefore the samples were dried and placed in between two NaBr windows. As background the empty windows were measured. The measurements were done under nitrogen atmosphere.

## Figures and Tables

**Table 1:** Specific amounts of chemicals used for the Synthesis of the TPM-grafted silica particles.

Name	Ethanol	NH_3_ (A)	TEOS	NH_3_ (B)	TPM
	mL	mL	mL	mL	mL
Si-1	200	15	5	10	1
Si-2	450 *	20	7.5	15	2
Si-3	750	35	5	10	1

* Here 99% ethanol was use. Tetraethylorthosilicate (TEOS); 3-(trimethoxysilyl)-propyl-methacrylate (TPM).

**Table 2:** Amounts of the chemicals used for the synthesis of SiPn-3 (a–c). Methylene-bis-acrylamide (BIS).

Chemical	Nipam mmol	BIS mmol
First addition	17.67	0.57
Second addition	17.67	0.57
Third addition	17.67	0.65
sum	53.01	1.79

## Supplementary Materials

The following are available online at www.mdpi.com/2310-2861/3/3/34/s1, Figure S1: TGA measurements (a) and its derivative (b) of Si-2, SiTPM-2, SiPN-1, SiPN-2 and SiPn3, Table S1: Weight loss of the sample:Si2, SiTPM-2, SiPN-1, SiPN-2 and SiPn3 within the temperature range 25–150 °C (region 1) and within the temperature range 150–500 °C (region 2) and residue at 500 °C. In the first region the weight loss corresponds to the solvent evaporation. In the second region the weight loss corresponds to the thermal decomposition of the organic content.
