# Supplementary material for: Tuning the Size of Thermoresponsive Poly(N-Isopropyl Acrylamide) Grafted Silica Microgels
Source: Gels. 2017 Sep 17;3(3):34. doi: 10.3390/gels3030034 (PMC6318582; doi:10.3390/gels3030034)
Supplement: Supplementary file 1 [file gels-03-00034-s001.pdf]

gels

MDPI

*Article*

# Tuning the Size of Thermoresponsive Poly(*N*-Isopropyl Acrylamide) Grafted Silica Microgels

**Nils Nun, Stephan Hinrichs, Martin A. Schroer, Dina Sheyfer, Gerhard Grübel and Birgit Fischer**

## 1. TGA measurements

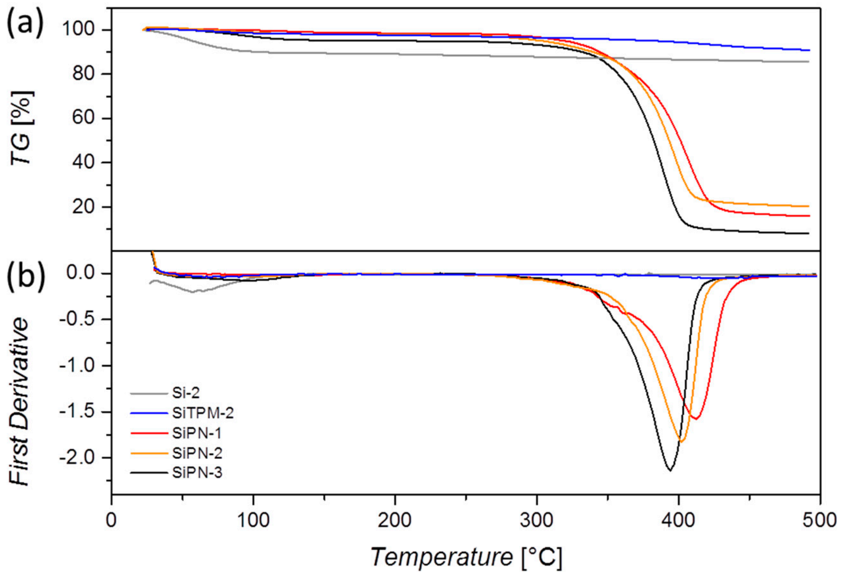


**Figure S1.** TGA measurements (**a**) and its derivative (**b**) of Si-2, SiTPM-2, SiPN-1, SiPN-2 and SiPn3.

**Table S1.** Weight loss of the sample:Si2, SiTPM-2, SiPN-1, SiPN-2 and SiPn3 within the temperature range 25-150 °C (region 1) and within the temperature range 150-500 °C (region 2) and residue at 500 °C. In the first region the weight loss corresponds to the solvent evaporation. In the second region the weight loss corresponds to the thermal decomposition of the organic content.

| **name** | **1st region** | | | **2nd region** | | | **Residue at 500°C [%]** |
|---|---|---|---|---|---|---|---|
| | Range [°C] | Peak [°C] | Weight loss [%] | Range [°C] | Peak [°C] | Weight loss [%] | |
| Si-2 | 25-150 | 56.8 | 10.3 | 150-500 | - | 3.9 | **85.8** |
| SiTPM-2 | 25-150 | - | 2.0 | 150-500 | 422.3 | 7.2 | **90.8** |
| SiPn-1 | 25-150 | - | 1.4 | 150-500 | 412.3 | 83.0 | **15.6** |
| SiPn-2 | 25-150 | - | 2.9 | 150-500 | 401.3 | 77.8 | **19.3** |
| SiPn-3 | 25-150 | 99.3 | 5.6 | 150-500 | 394.3 | 87.2 | **7.2** |
